# Interplay between FGFR2b‐induced autophagy and phagocytosis: role of PLCγ‐mediated signalling

**DOI:** 10.1111/jcmm.13352

**Published:** 2017-10-10

**Authors:** Monica Nanni, Danilo Ranieri, Salvatore Raffa, Maria Rosaria Torrisi, Francesca Belleudi

**Affiliations:** ^1^ Laboratory affiliated to Istituto Pasteur Italia – Fondazione Cenci Bolognetti Department of Clinical and Molecular Medicine Sapienza University of Rome Rome Italy; ^2^ S. Andrea University Hospital Rome Italy

**Keywords:** FGFR2, FGFR2b, PLCγ, autophagy, phagocytosis, keratinocytes

## Abstract

Signalling of the epithelial splicing variant of the fibroblast growth factor receptor 2 (FGFR2b) induces both autophagy and phagocytosis in human keratinocytes. Here, we investigated, in the cell model of HaCaT keratinocytes, whether the two processes might be related and the possible involvement of PLCγ signalling. Using fluorescence and electron microscopy, we demonstrated that the FGFR2b‐induced phagocytosis and autophagy involve converging autophagosomal and phagosomal compartments. Moreover, the forced expression of FGFR2b signalling mutants and the use of specific inhibitors of FGFR2b substrates showed that the receptor‐triggered autophagy requires PLCγ signalling, which in turn activates JNK1 *via* PKCδ. Finally, we found that in primary human keratinocytes derived from light or dark pigmented skin and expressing different levels of FGFR2b, the rate of phagocytosis and autophagy and the convergence of the two intracellular pathways are dependent on the level of receptor expression, suggesting that FGFR2b signalling would control *in vivo* the number of melanosomes in keratinocytes, determining skin pigmentation.

## Introduction

The fibroblast growth factor receptors (FGFRs) are receptor tyrosine kinases (RTKs) regulating several cellular processes such as proliferation, differentiation, migration and survival 1, 2. FGF binding to FGFRs induces the receptor dimerization and transphosphorylation, which activates different intracellular signalling pathways. The main downstream substrates of FGFRs are the phospholipase Cγ (PLCγ), which associates with FGFRs in a ligand‐inducible manner *via* specific receptor tyrosine residues, and the FGFR substrate 2 (FRS2). FRS2 phosphorylation leads to subsequent activation of both RAS/mitogen‐activated protein kinase (MAPK) and phosphoinositide 3‐kinase (PI3K)/AKT signalling pathways 3. Other pathways may be also activated downstream FGFRs, including the Jun N‐terminal kinases (JNKs) pathway 4, which can be activated downstream RAS by the MAPKKs, MKK4 and MKK7 5, or *via* protein kinase C delta (PKCδ) 6, 7.

FGFRs are expressed on different tissues, where the specific alternative splicing of the IgIII domain in FGFR1–3 produces IIIb epithelial and IIIc mesenchymal isoforms determining the ligand specificity 1, 2. The FGFR2b/KGFR is exclusively expressed in epithelial cells 8 and can be activated by the stimulation with its specific ligand FGF7/KGF 9. We have recently demonstrated that FGFR2b expression and its signalling trigger autophagy in human keratinocytes, and the use of specific inhibitors indicated that this effect is PI3K/AKT/mTOR‐independent 10. Moreover, the selective block of autophagosome fusion with lysosomes demonstrated that FGFR2b‐mediated signalling stimulates the autophagosome assembly, but also their turnover upon a prolonged autophagic stimulus 10. In addition, recent findings from our group showed that the expression of the E5 oncoprotein of human papillomavirus type 16 (HPV16 E5) is able to inhibit the FGF7‐induced autophagy through down‐regulation of FGFR2b 11.

A functional and molecular link between autophagy and phagocytosis appears to exist, at least in macrophages. In fact, it has been demonstrated that autophagy induction promotes the engulfment of phagosomes containing bacteria in autophagosomes to allow pathogen elimination 12, 13. However, other studies have shown that the induction of autophagy leads to down‐modulation of the phagocytosis of yeast particles in murine macrophages 14 and that the phagocytosis is enhanced in macrophages from ATG7‐deficient mice 15. In addition, TLR signalling during phagocytosis induces a rapid recruitment of BECN1 and microtubule‐associated protein 1 light chain 3 (LC3) to the phagosomes, apparently without the formation of conventional autophagosomes, in the process called LC3‐associated phagocytosis (LAP) 16. Several other ATG proteins are also required for different steps of LAP, including the phagosome maturation 16, 17.

As we have previously demonstrated that FGF7‐dependent activation of FGFR2b and its downstream PLCγ signalling trigger phagocytosis and melanosome uptake in human keratinocytes 18, here we investigated the possible interplay between FGFR2b‐induced autophagy and phagocytosis and the involvement of PLCγ signalling also in the receptor‐mediated autophagic process.

## Materials and methods

### Cells and treatments

The human keratinocyte cell line HaCaT 19 was cultured in Dulbecco's modified Eagle's medium (DMEM), supplemented with 10% foetal bovine serum (FBS) plus antibiotics. Primary cultures of human keratinocytes derived from light healthy skin (light HKs) were obtained from patients attending the Dermatology Unit of the Sant'Andrea Hospital of Rome; all patients were extensively informed and their consent for the investigation was given and collected in written form in accordance with guidelines approved by the management of the Sant'Andrea Hospital. Primary keratinocytes were isolated and cultured as previously described 20. Primary cultures of darkly pigmented HKs (dark HKs) were purchased from Cascade Biologics (Portland, OR, USA).

Cells were transiently transfected with pCI‐neo empty vector (HaCaT pCI‐neo), with pCI‐neo expression vector containing human FGFR2b (HaCaT FGFR2b WT) or a kinase‐negative mutant FGFR2b Y656F/Y657F (HaCaT FGFR2b kin^−^) 21 or a signalling mutant FGFR2b Y769F (HaCaT FGFR2b Y769F) 22. Alternatively, cells were transiently transfected with pEGFP‐C2 expression vector containing LC3 (engineered by Dr. Fimia, National Institute for Infectious Diseases IRCCS ‘L. Spallanzani’, Rome, Italy; and kindly provided by Prof. Francesco Cecconi, Tor Vergata University of Rome, Italy). jetPEI™ DNA Transfection Reagent (Polyplus‐transfection, New York, NY, USA, 10‐40) was used for transfection of HaCaT cells, while FuGENE HD Transfection reagent (Promega, Madison, WI, USA, E2311) was used for transfection of HKs.

For RNA interference and ULK1 or Rubicon silencing, HaCaT cells were transfected with ULK1 small interfering RNA (ULK1 siRNA) (Santa Cruz Biotechnology, Santa Cruz, CA, USA, sc‐44182), or with Rubicon small interfering RNA (Rubicon siRNA) (Santa Cruz Biotechnology, sc‐78326) or with an unrelated siRNA as a control (control siRNA) (Santa Cruz Biotechnology, sc‐37007), using Lipofectamine 2000 Transfection Reagent (Invitrogen, Carlsbad, CA, USA, 11668‐019) according to the manufacturer's protocol.

For stimulation of growth factors, cells were serum‐starved and incubated with FGF7 (Upstate Biotechnology, Lake Placid, NY, USA, 01‐118) 100 ng/ml for 24 hrs at 37°C.

For inhibition of FGFR2b activity, cells were preincubated with a specific FGFR tyrosine kinase inhibitor, SU5402 (25 μM; Calbiochem, Nottingham, UK, 572630), for 1 hr before treatment with the growth factor.

To inhibit AKT or ERK or JNK or PKCδ, cells were respectively incubated with the specific AKT inhibitor 1L‐6‐hydroxymethyl‐chiro‐inositol 2‐(R)‐2‐O‐methyl‐3‐O‐octadecylcarbonate (1 μM; Calbiochem, 124005) or with the specific MEK1/2 inhibitor PD0325901 (1 μM; Sigma‐Aldrich, St. Louis, MO, USA, PZ0162) or with the specific JNK inhibitor SP600125 (50 μM; Sigma‐Aldrich, S5567) or with the specific PKCδ inhibitor rottlerin (0.5 μM; Calbiochem, 557370) for 1 hr at 37°C before treatment with FGF7 in the presence of each inhibitor.

To irreversibly block the fusion between autophagosomes and lysosomes, HaCaT cells were incubated with bafilomycin A1 (20 nM; Sigma‐Aldrich, B 1793) 23 for 3 hrs at 37°C after treatment with FGF7 in the presence of the inhibitor.

To analyse the uptake of beads in keratinocytes, cells were incubated with red fluorescent microspheres 0.5 and 1 μm in diameter (FluoSpheres; Molecular Probes, Eugene, OR, USA, F8812 and F8821) at the concentration of 72 × 10^7^ particles/ml and 36 × 10^7^ particles/ml for 4 hrs, respectively. These microspheres are inert latex fluorescent spherical particles loaded with a red (580/605) fluorescent dye which show little or no photobleaching.

To evaluate the effects of FGFR2b activation on the phagocytic ability, the uptake was performed in the presence of 100 ng/ml FGF7.

### Immunofluorescence

Cells, grown on coverslips and incubated with or without FGF7 as above, were fixed with 4% paraformaldehyde (Electron Microscopy Sciences, Hatfield, PA, USA, 157‐8) in PBS for 30 min. at 25°C followed by treatment with 0.1 M glycine (Sigma‐Aldrich, 50046) for 20 min. at 25°C and with 0.1% Triton X‐100 for an additional 5 min. at 25°C to allow permeabilization. Cells were then incubated for 1 hr at 25°C with the following primary antibodies: the rabbit polyclonal anti‐Bek (1:50 in PBS; C‐17; Santa Cruz Biotechnology, sc‐122) directed against the intracellular portion of FGFR2 isoforms and mouse monoclonal anti‐LAMP‐2 (CD170b; BD Biosciences, San Josè, CA, USA, 55803). The primary antibodies were visualized using goat anti‐rabbit IgG‐Texas Red (1:200 in PBS; Jackson ImmunoResearch Laboratories, West Grove, PA, USA, 111‐075‐144) and goat antimouse IgG‐Alexa Fluor 350 (1:20 in PBS; Life Technologies, Carlsbad, CA, USA, A‐11045) for 30 min. at 25°C. Nuclei were stained with DAPI (1:1000 in PBS; Sigma‐Aldrich, D9542). Coverslips were finally mounted with Mowiol (Sigma‐Aldrich, 81381) for observation.

Fluorescence signals were analysed by scanning cells in a series of sequential sections with an ApoTome System (Zeiss, Oberkochen, Germany, 000000‐1189‐776); image analysis was performed by the Axiovision software (Zeiss, 410130‐9850‐000), and 3D reconstruction of a selection of three central optical sections is shown in each figure. Quantitative analysis of EGFP‐LC3‐positive dots per cell was performed analysing 100 cells for each sample in five different microscopy fields from three different experiments. The EGFP‐LC3‐positive dots correspond to the amount of the autophagosomes. In fact, the punctate staining of EGFP‐LC3 represents the autophagosome membrane‐associated form of LC3 (LC3‐II), derived from the lipidation of the LC3 cytosolic form (LC3‐I). Quantitative analysis of the bead uptake was performed by counting the number of internalized beads in 100 cells for each condition, randomly taken from 10 microscopic fields in three different experiments. Quantitative analysis of the extent of colocalization of beads with EGFP‐LC3 and LAMP‐2 was performed by the analysis of 100 cells for each sample in five different fields randomly taken from three independent experiments and using the KS300 3.0 Image Processing System (Zeiss, 000000‐1020‐771).

Results are expressed as mean values ± S.E. *P* values were calculated using Student's *t*‐test, and significance level was defined as *P* < 0.05.

### Western blot analysis

Cells were lysed, and total protein was resolved by SDS‐PAGE and transferred to reinforced nitrocellulose as previously described 10. The membranes were blocked with 5% non‐fat dry milk (Bio‐Rad Laboratories, Hercules, CA, USA, 170‐6404) in PBS/0.1% Tween‐20 (Bio‐Rad, 170‐6531) or with 3% BSA (Sigma‐Aldrich, A7030) in PBS/0.1% Tween‐20, and incubated with anti‐Bek polyclonal antibodies (C17; Santa Cruz Biotechnology, sc‐122), anti‐LC3 polyclonal antibodies (MBL, Woburn, MA, USA, PD014), anti‐P‐p44/42 MAPK (P‐ERK1/2) polyclonal antibodies (Thr202/Tyr204, Cell Signaling Technology, Beverly, MA, USA, 9101S), anti‐p‐mTOR monoclonal antibody (Ser 2448, Cell Signaling, 5536), anti‐p‐AKT polyclonal antibodies (Ser 473, Cell Signaling, 9271), anti‐p‐JNK monoclonal antibody (G9, Cell Signaling, 9255S), anti‐p‐PKCδ polyclonal antibodies (Tyr‐155; Santa Cruz Biotechnology, sc‐23770‐R), anti‐p‐PKCδ monoclonal antibody (Ser 645, Abcam, Cambridge, UK, ab108972), anti‐ULK1 polyclonal antibodies (H‐240; Santa Cruz Biotechnology, sc‐33182), and anti‐Rubicon monoclonal antibody (D9F7, Cell Signaling, 8465), followed by enhanced chemiluminescence detection (ECL, Amersham, Arlington Heights, IL, USA, 34080). The membranes were rehydrated by washing in PBS/Tween‐20, stripped with 100 mM mercaptoethanol and 2% SDS for 30 min. at 55°C and probed again with anti‐mTOR monoclonal antibody (7C10, Cell Signaling, 2983), anti‐AKT polyclonal antibodies (H‐136; Santa Cruz Biotechnology, sc‐8312), anti‐p44/42 MAPK (ERK1/2) polyclonal antibodies (137F5, Cell Signaling, 4695S), anti‐JNK polyclonal antibodies (Cell Signaling, 9252S), anti‐PKCδ polyclonal antibodies (C‐20; Santa Cruz Biotechnology, sc‐937), anti‐β‐actin monoclonal antibody (Sigma‐Aldrich, A5441) or anti‐α‐tubulin polyclonal antibodies (Cell Signaling, 2148S) to estimate the protein equal loading. Densitometric analysis was performed with Quantity One program (Bio‐Rad). Results from three different experiments were normalized, expressed as fold increase with respect to the control value and reported as mean values.

### Transmission electron microscopy

HaCaT cells stimulated with FGF7 for 24 hrs and treated with red fluorescent beads for the last 4 hrs as above were washed three times in PBS and fixed with 2% glutaraldehyde (Electron Microscopy Science, 16300) in PBS for 2 hrs at 4°C. Samples were postfixed with 1% osmium tetroxide in veronal acetate buffer (pH 7.4) for 1 hr at 25°C, stained with uranyl acetate (5 mg/ml) for 1 hr at 25°C, dehydrated in acetone and embedded in Epon 812 (EMbed 812, Electron Microscopy Science). Ultrathin sections were examined unstained or poststained with uranyl acetate and lead hydroxide, under a Morgagni 268D transmission electron microscope (FEI, Hillsboro, OR, USA) equipped with a MegaView II charge‐coupled device camera (SIS, Soft Imaging System GmbH, Munster, Germany) and analysed with AnalySIS software (SIS).

## Results

### FGFR2b‐mediated phagocytosis and autophagy are convergent pathways regulated by PLCγ signalling

**Figure 1:**
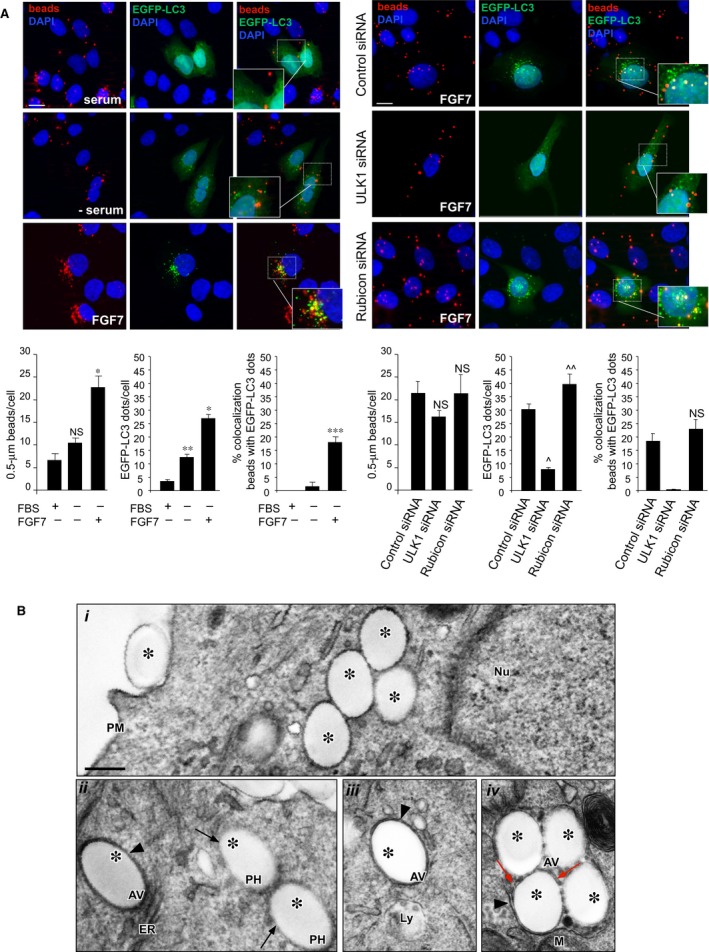
Presence of internalized beads in autophagosomes after FGF7 stimulation. (**A**) HaCaT cells were transiently transfected with pEGFP‐C2‐LC3 construct (left panels) or cotransfected with LC3 construct and ULK1 siRNA, Rubicon siRNA or an unrelated siRNA as control (right panels). Cells were then serum‐starved and stimulated with FGF7 for 24 hrs and with inert latex red fluorescent beads for the last 4 hrs. Cell nuclei were stained with DAPI. Quantitative fluorescence analysis shows that while in cells singly transfected with EGFP‐LC3 the serum deprivation only induces an increase in the number of EGFP‐LC3‐positive dots per cell, FGF7 stimulation enhances either the number of EGFP‐LC3‐positive dots or fluorescent bead uptake per cell (left panels); a partial colocalization between LC3 and fluorescent beads is detectable only in response to FGF7 (left panels). ULK1 depletion reduces, while Rubicon depletion further increases the number of EGFP‐LC3‐positive dots compared to control cells (right panels); the colocalization between EGFP‐LC3 and the fluorescent beads in response to FGF7 is significantly reduced only by ULK1 depletion (right panels). None of the siRNAs used significantly affects beads uptake (right panels). The quantitative analysis was performed as described in Materials and Methods, and results are expressed as mean values ± S.E. Student's *t‐*test was performed, and significance levels were defined as *P *<* *0.05: *P <* *0.001 *versus* the corresponding FGF7‐unstimulated cells, **P <* *0.001 *versus* the corresponding serum‐cultured cells, ***P <* *0.01 *versus* the corresponding FGF7‐unstimulated cells, ^*P* < 0.001 *versus* HaCaT control siRNA cells, ^^*P* < 0.05 *versus* HaCaT control siRNA cells, NS (not significant) *versus* the corresponding serum‐cultured cells and *versus* HaCaT control siRNA cells. Bar: 10 μm. (**B**) Ultrastructural analysis of HaCaT cells stimulated with FGF7 in the presence of beads as described above. Single (*i*,* ii*,* iii,* asterisks) and clustered beads (*iv*, asterisks) were visible in either single‐membrane (*ii,* arrows) or double‐membrane vacuoles (*ii*,* iii, iv*, arrowheads) corresponding to phagosomes and autophagosomes, respectively. The clustered beads enclosed in a double‐membrane vacuole also appeared singly surrounded by a single membrane (*iv*, red arrows). AV: auophagic vacuole; ER: endoplasmic reticulum; Ly: lysosome; M: mitochondrion; Nu: nucleus; PH: phagosome; PM: plasma membrane. Bar: 0.25 μm.

We have recently demonstrated that FGFR2b expression and signalling trigger both autophagy 10 and phagocytosis 18, 20 in human keratinocytes. To assess the existence of a possible interrelationship between the two processes, when induced by FGF7 binding to the receptor, and to ascertain whether they could involve converging autophagosomal and phagosomal compartments, we took advantage of the use of an *in vitro* model of bead uptake widely used to study the phagocytic ability of epidermal keratinocytes 24, 25, 26. The human keratinocyte HaCaT cell line 19 was transiently transfected with the construct EGFP of the widely accepted marker for autophagosomes LC3 27, 28 (pEGFP‐C2‐LC3), serum‐starved and stimulated with FGF7 for 24 hrs. For the last 4 hrs of the treatment, cells were incubated also with inert latex red fluorescent beads 0.5 μm in diameter, whose size is comparable to that of melanosomes 18, 20. Concentrations of both FGF7 and beads, as well as the times of treatments, were selected based on our published results 18, 20, 29. Cells were fixed and permeabilized, and nuclei were stained with DAPI. Quantitative fluorescence analysis, performed as reported in Materials and Methods, showed that while the serum deprivation, which is a well‐known autophagic stimulus, only increased the number of the LC3‐positive dots per cell, as expected the treatment with FGF7 increased both the bead uptake 18, 20 (Fig. 1A, left panels) and the amount of EGFP‐LC3‐positive dots per cell 10 (Fig. 1A, left panels). In addition, only after FGF7 stimulation the internalized beads partially colocalized with LC3 (18%) (Fig. 1A, left panels)**.** To confirm that the choice of 0.5‐μm‐diameter beads is the most appropriate for our study, parallel experiments were performed with larger beads (1 μm diameter). The results showed that, even if both bead uptake and the number of LC3‐positive dots per cell were significantly increases by FGF7 stimulation, the amount of the internalized beads was reduced, in particular following FGF7 treatment (Fig. S1), when compared to that observed using 0.5‐μm‐sized beads (see Fig. 1A, left panels). In addition, very poor colocalization between the beads and the LC3‐positive dots was observed (Fig. S1), suggesting that the use of smaller size beads was the most suitable condition to highlight the effect of FGF7 in inducing the convergence between phagocytosis and autophagy.

Interestingly, in the experiments performed with 0.5‐μm beads, the not so tight colocalization and the presence of distal formation of autophagosomes (LC3‐positive dots which did not colocalize with beads) strongly indicated that FGF7 induces LC3 association with canonical newly formed autophagic vesicles and not directly with the membrane of all the nascent phagosomes, as occurs during LAP. This is consistent with the fact that inert beads are not able to activate LAP 30. To further confirm that LC3 was mainly associated with autophagosomes and not with LAP‐engaged phagosomes (LAPosomes), the stimulation with FGF7 was performed in cells in which the canonical autophagy was inhibited through ULK1 protein depletion by specific siRNA transfection. ULK1 is a component of the ULK initiation complex required for canonical autophagy 31, but not for LAP 32, and its depletion provides a useful tool for distinguishing between the two processes 33. On the other hand, to obtain specific inhibition of LAP, we performed the depletion of Rubicon, which activates the UVRAG‐containing class III PI(3)K complex exclusively required for LAPosome formation 30 and represses the canonical autophagy inhibiting the activity of Beclin 1–PI(3)K complex 34, 35. The results showed that silencing of ULK1 did not affect bead uptake, but significantly decreased the number of EGFP‐LC3‐positive dots per cell, as well as the colocalization between these dots and the fluorescent beads (Fig. 1A, right panels). These results further suggested that upon FGF7 stimulation, LC3 associates with nascent autophagosomes but not with LAPosomes and that the colocalization between LC3 and the fluorescent beads observed in untransfected cells or in cells transfected with control siRNA possibly derives from the confluence between distinct phagosomes and autophagosomes. Consistent with this possibility, Rubicon silencing resulted in an opposite effect, enhancing the number of LC3‐positive dots per cell and increasing, although not significantly, their colocalization with fluorescent beads (Fig. 1A, right panels). The efficient depletion of the ULK1 or Rubicon proteins was verified in siRNA‐transfected cells through Western blot analysis (Fig. S2). To further confirm that the LC3‐positive dots, which colocalize with fluorescent beads, correspond to distinct autophagosomal structures, electron microscopy studies were performed in HaCaT cells stimulated with beads in the presence of FGF7 as above. Ultrastructural examination revealed the presence of single (Fig. 1B *i*,* ii* and *iii,* asterisks) and clustered beads (Fig. 1B *iv*, asterisks) in either single‐membrane (Fig. 1B *ii*, arrows) or more electron‐dense double‐membrane (Fig. 1B *ii*,* iii* and *iv*, arrowheads) vacuoles corresponding to phagosomes and autophagosomes, respectively. Interestingly, the clustered beads collectively enclosed in a double‐membrane vacuole also appeared singly surrounded by a single membrane (Fig. 1B *iv,* red arrows), suggesting that the autophagosome could be generated and closed around phagosomes containing the bead. Several clustered beads are also visible in vacuoles ultrastructurally recognizable as lysosomes (data not shown), confirming that the final fate for the engulfed beads is the degradative compartment. These results clearly suggest that in response to FGF7, part of the phagosomes containing the engulfed beads are isolated in new‐forming autophagosomes that possibly mediate their targeting to lysosomes.

**Figure 2:**
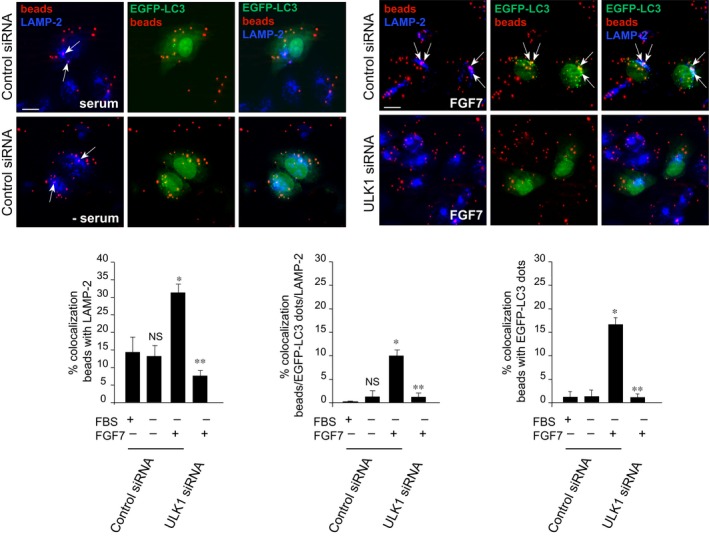
Autophagy improves phagosome fusion with lysosomes in response to FGF7. HaCaT cells were transiently cotransfected with EGFP‐LC3 and with ULK1 siRNA, to inhibit autophagy, or with an unrelated siRNA as control, and then serum‐starved and stimulated with FGF7 as above. Quantitative immunofluorescence analysis performed with anti‐LAMP‐2 antibody shows that in unrelated siRNA‐transfected samples, the low colocalization between fluorescent beads and LAMP‐2, which is detectable in cells grown in serum‐added or in serum‐deprived medium (left panels, arrows), is significantly increased by FGF7 treatment (right panels, top, arrows); triple colocalization between LC3, beads and LAMP‐2 is visible only in FGF7‐stimulated cells (right panels, top, arrows). All the FGF7‐induced signal overlaps were strongly impaired by ULK1 silencing (right panels, bottom). The quantitative analysis and Student's *t‐*test were performed as above: *P <* *0.001 *versus* the corresponding FGF7‐unstimulated cells; **P <* *0.001 *versus* the corresponding control siRNA cells; NS *versus* the corresponding serum‐cultured cells.

To verify whether FGF7‐induced autophagy actually contributes to increase the efficiency of phagosome fusion with lysosomes, triple‐immunofluorescence approaches were performed in HaCaT cells transiently cotransfected with EGFP‐LC3 and ULK1 siRNA, to inhibit the autophagic process. Alternatively, cells were cotransfected with an unrelated siRNA as control. Upon FGF7 stimulation performed as above, cells were fixed and permeabilized and the immunostaining with anti‐LAMP‐2 antibody was performed to visualize the lysosomal compartment. Quantitative analysis showed that in cells transfected with the unrelated siRNA, the low colocalization between fluorescent beads and LAMP‐2 visible in cells grown in serum‐added (Fig. 2, left panels, top, arrows) or in serum‐deprived medium (Fig. 2, left panels, bottom, arrows) was significantly increased by FGF7 stimulation (Fig. 2, right panels, top, arrows), suggesting that the phagosomal fusion with lysosomes was improved. In addition, the appearance of a triple colocalization between LC3, beads and LAMP‐2 only in response to FGF7 (Fig. 2, right panels, top, arrows) clearly indicated that this stimulation is able to drive phagosomes and autophagosomes to converge and reach the degradative compartment. Interestingly, the signal overlaps observed in FGF7‐stimulated cells were strongly impaired by ULK1 silencing (Fig. 2, right panels, bottom), suggesting that the autophagic process is required for the improved efficiency of the fusion between phagosomes and lysosomes induced by FGF7.

**Figure 3:**
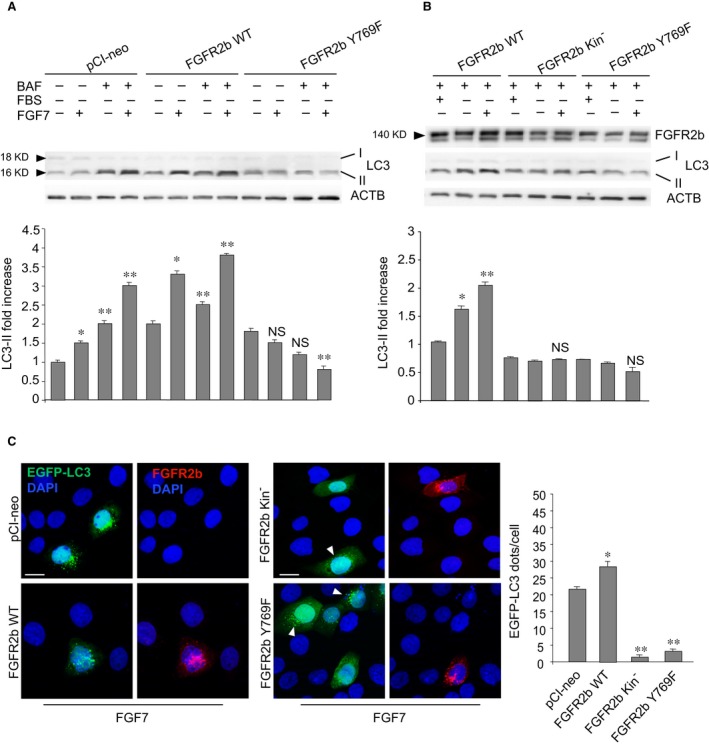
The expression of the PLCγ signalling mutant FGFR2b Y769F inhibits FGF7‐induced autophagy. (**A, B**) HaCaT cells were transiently transfected with the FGFR2b WT or with the Y769F FGFR2b mutant. The transfection with the kinase‐negative Y656F/Y657F FGFR2b or with pCI‐neo empty vector was performed as control. Upon transfection, cells were serum‐starved and stimulated with FGF7 in the presence or absence of bafilomycin A1 for the last 3 hrs. (**A**) Western blot analysis shows that the increase in the levels of LC3‐II upon FGF7 stimulation, visible in HaCaT pCI‐neo and even more in HaCaT FGFR2b WT cells, is further enhanced by bafilomycin A1; independent from the presence of this drug, FGFR2b Y769F cells appear not responsive to FGF7. (**B**) FGFR2b Y769F expression, as well as FGFR2b kin^−^ expression, inhibits the increase in LC3‐II levels induced by FGF7 in FGFR2b WT cells. The equal loading was assessed using anti‐ACTB antibody. For the densitometric analysis, the values from three independent experiments were normalized, expressed as fold increase and reported in graph as mean values ± S.D. Student's *t‐*test was performed, and significance levels were defined as *P *<* *0.05: (**A**) *P < 0.05 *versus* the corresponding FGF7‐unstimulated cells, **P < 0.05 *versus* the corresponding bafilomycin‐untreated cells, NS *versus* the corresponding FGF7‐unstimulated cells and *versus* the corresponding bafilomycin‐untreated cells. (**B**) *P <* *0.05 *versus* the corresponding serum‐cultured cells, **P <* *0.05 *versus* the corresponding FGF7‐unstimulated cells, NS *versus* the corresponding FGF7‐unstimulated cells. (**C**) HaCaT cells were transiently cotransfected with pEGFP‐C2‐LC3 construct and with the FGFR2b WT, with the Y769F FGFR2b mutant, with the kinase‐negative Y656F/Y657F FGFR2b or with pCI‐neo empty vector and stimulated with FGF7 as above. Immunofluorescence was performed with anti‐FGFR2b polyclonal antibodies to visualize transfected FGFR2b WT or mutants. Upon FGF7 treatment, the number of LC3‐positive dots per cell is reduced in HaCaT EGFP‐LC3/FGFR2b Y769F cells, as well as in HaCaT EGFP‐LC3/FGFR2b kin^−^ cells, compared to the surrounding cells not showing detectable receptor mutant overexpression (arrowheads), or to HaCaT EGFP‐LC3/pCI‐neo and HaCaT EGFP‐LC3/FGFR2b WT cells. Different from the kinase‐negative dead mutant, both FGFR2b WT and FGFR2b Y769F signals appear internalized upon FGF7 stimulation. The quantitative analysis and Student's *t‐*test were performed as above: *P <* *0.05 *versus* HaCaT pCI‐neo, **P <* *0.001 *versus* HaCaT FGFR2b WT cells. Bar: 10 μm.

Being PLCγ activation/phosphorylation required for FGFR2b‐mediated phagocytosis 18, to investigate the role of this signalling in FGF7‐induced autophagy we transiently transfected HaCaT cells with a FGFR2b signalling mutant in which the tyrosine 769, required for PLCγ binding and activation 22, 36, has been substituted by phenylalanine (Y769F) 22. The transfection with FGFR2b wild‐type (HaCaT FGFR2b WT) or with pCI‐neo empty vector (HaCaT pCI‐neo) was used as control. After transfection, cells were serum‐starved and stimulated with FGF7 for 24 hrs and the amount of LC3‐II was analysed by Western blot. To estimate whether PLCγ signalling shutdown could specifically impact on the autophagosome assembly induced by FGF7, we compared cells in which the autophagosome turnover was selectively blocked or not through the treatment with bafilomycin A1, an inhibitor of the vacuolar‐type H^+^‐ATPase (v‐ATPase) able to block the fusion of autophagosomes with lysosomes 23. Different from other agents, including monensin or chloroquine, bafilomycin A1 does not activate LC3 association with single intracellular membranes, such as phagosomes, as occurs during LAP 37. The results showed that, as previously demonstrated 10, the increase in LC3‐II levels in response to FGF7, evident in pCI‐neo cells (Fig. 3A) and even more in FGFR2b WT cells (Fig. 3A), was further increased by the presence of bafilomycin A1 (Fig. 3A), confirming that FGF7 acts inducing autophagosome formation. In contrast, independent from the treatment with bafilomycin A1, FGFR2b Y769F‐expressing cells did not display an increase in LC3‐II in response to FGF7 (Fig. 3A). Thus, PLCγ signalling shutdown is sufficient to repress the autophagosome assembly induced by FGF7. In agreement with this conclusion, the effect observed in FGFR2b Y769F cells appeared comparable to that detected in cells transfected with a Y656F/Y657F FGFR2b kinase dead mutant (HaCaT FGFR2b kin^−^) 21 (Fig. 3B). The impact of PLCγ signalling on FGF7‐induced autophagy was also investigated by fluorescence approaches. To this aim, HaCaT cells were alternatively cotransfected with pEGFP‐C2‐LC3 construct and pCI‐neo empty vector, FGFR2b WT, FGFR2b kin^−^ or the FGFR2b Y769F. Cells were then treated with FGF7 as above, fixed and permeabilized, and nuclei were stained with DAPI. Quantitative immunofluorescence analysis performed with anti‐FGFR2b polyclonal antibodies, to visualize transfected FGFR2b WT or mutants, showed that similar to FGFR2b kin^−^, FGFR2b Y769F overexpression induced a drastic reduction of the number of LC3‐positive dots compared to that observed in HaCaT EGFP‐LC3/pCI‐neo and in HaCaT EGFP‐LC3/FGFR2b WT cells (Fig. 3C) or in HaCaT EGFP‐LC3/FGFR2b Y769F and in EGFP‐LC3/FGFR2b kin^−^ surrounding cells not showing detectable receptor mutant overexpression (Fig. 3C, arrowheads). These results indicate that similar to that previously demonstrated for FGF7‐induced phagocytosis 18, the overexpression of FGFR2b Y769F or FGFR2b kin^−^ exerts an inhibitory effect also on FGF7‐induced autophagy. On the other hand, different from what was observed for the receptor kinase dead mutant (Fig. 3C), upon FGF7 stimulation both FGFR2b WT and FGFR2b Y769F signals appeared concentrated in intracellular dots (Fig. 3C), confirming that PLCγ signalling is not required for receptor internalization 22. Overall, our lines of evidence strongly suggest that the specific signalling downstream PLCγ is required for FGF7‐induced autophagosome formation and that its shutdown results in a significant inhibition of the process.

### FGFR2b‐induced autophagy requires PLCγ‐mediated phosphorylation/activation of JNK

**Figure 4:**
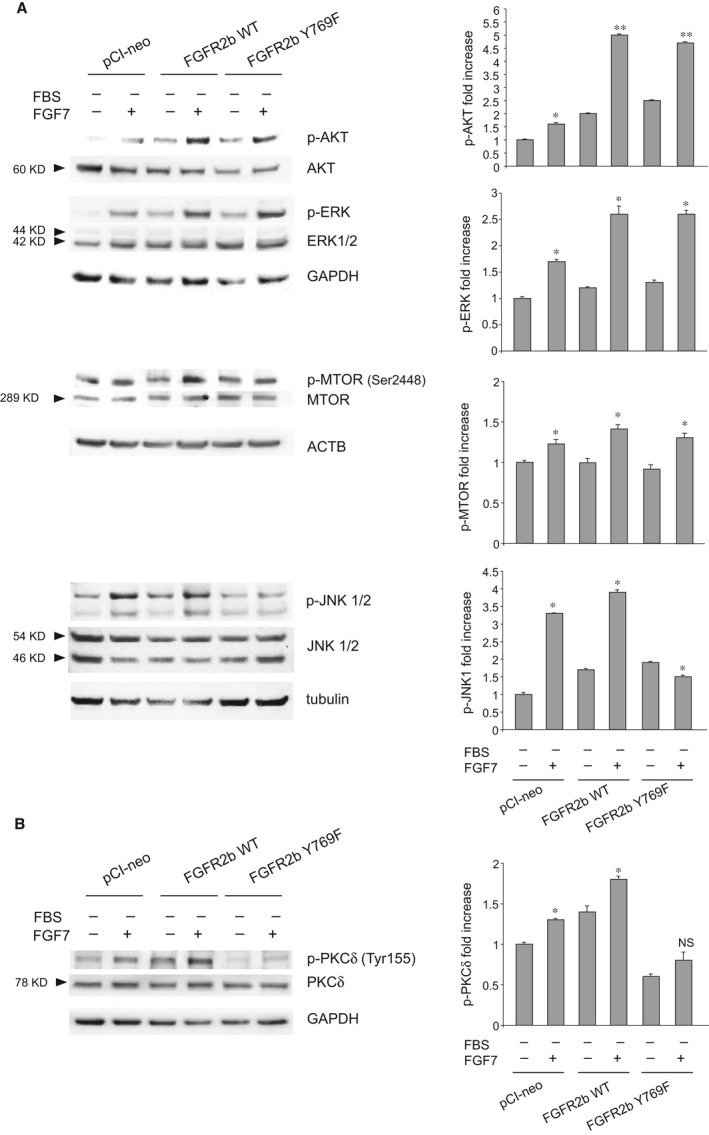
The inhibition of PLCγ signalling is accompanied by JNK pathway shutdown and PKCδ inactivation. (**A, B**) HaCaT pCI‐neo, HaCaT FGFR2b WT and HaCaT FGFR2b Y769F cells were serum‐starved and stimulated with FGF7 as above. Western blot analysis shows that in response to FGF7 stimulation, the phosphorylation of both JNK1 (**A**) and PKCδ, at the tyrosine 155 site (**B**), appears attenuated only in HaCaT FGFR2b Y769F cells, while MTOR (Ser 2448), AKT and ERK1/2 substrates (**A**) are highly phosphorylated in all cells. The equal loading was assessed using anti‐MTOR, anti‐AKT, anti‐ERK1/2, anti‐JNK1/2 and anti‐PKCδ antibodies. Housekeeping gene bands (ACTB, tubulin or GAPDH) are also shown. The densitometric analysis and Student's *t‐*test were performed as reported above: (**A**) *P <* *0.05 *versus* the corresponding FGF7‐unstimulated cells; **P <* *0.01 *versus* the corresponding FGF7‐unstimulated cells. (**B**) *P <* *0.05 *versus* the corresponding FGF7‐unstimulated cells; NS *versus* the corresponding FGF7‐unstimulated cells.

Autophagy can be controlled by several MTOR‐dependent or MTOR‐independent signalling pathways 38, 39. While it has been demonstrated that FGF2 negatively impacts on autophagy through the activation of AKT/MTOR signalling 40, 41, we have recently demonstrated that FGF7 increases autophagy through a not yet identified PI3K/AKT/MTOR‐independent pathway 10. Therefore, we focused our attention on the JNK1‐mediated signalling pathway, which is MTOR‐independent 39, and it has been recently identified as mainly involved in FGFR4‐mediated autophagy in chondrocytes 42. To assess the possible impact of the JNK1 pathway in FGF7‐mediated autophagy, and to establish whether and how PLCγ is involved in the activation of the JNK1 pathway, we compared the levels of phosphorylation/activation of this kinase and of other important FGFR2b signalling substrates involved in the control of autophagy, such as ERK1/2, in HaCaT pCI‐neo, HaCaT FGFR2b WT cells and HaCaT FGFR2b Y769F cells. Both AKT and MTOR phosphorylation was checked to further confirm the independence of FGF7‐mediated autophagy from the canonical PI3K/AKT/MTOR regulating pathway. Western blot analysis showed that upon FGF7 stimulation, all the checked substrates appeared phosphorylated in HaCaT pCI‐neo and HaCaT FGFR2b WT cultures, indicating that in these cells all the signalling pathways downstream FGFR2b are activated (Fig. 4A). In particular, the phosphorylation/activation of MTOR at serine 2448 (Ser2448) site (Fig. 4A) further confirmed the independence of FGF7‐induced autophagy by this signalling pathway. More interestingly, in HaCaT FGFR2b Y769F cells AKT, MTOR and ERK1/2 appeared normally phosphorylated, while JNK1 phosphorylation at threonine 183 and tyrosine 185 sites (Thr183/Tyr185) was significantly reduced (Fig. 4A). These lines of evidence indicated that PLCγ recruitment to FGFR2b and its consequent phosphorylation are required for an efficient activation of the JNK1‐mediated signalling pathway. JNK1 pathway is canonically activated by several MAP3 kinases, which in turn activate MKK4 and MKK7 that induce JNK phosphorylation 5. However, it has been recently demonstrated that PKCδ is the upstream substrate mainly responsible for the activation of the JNK1 pathway, during hypoxia‐mediated autophagy 7, 43. PKCδ is a substrate which is activated *via* PLCγ 44 also upon the activation of FGFRs, including FGFR2 45. To establish the relevance of PKCδ activation in the triggering of FGF7‐mediated autophagy, we compared its phosphorylation levels at the tyrosine 155 site in cells alternatively overexpressing FGFR2b or FGFR2b Y769F or pCI‐neo as control. In fact, Tyr 155 is one of the crucial residues whose phosphorylation is required for PKCδ upon different stimuli. Western blot analysis clearly demonstrated that in agreement with the observed attenuation of JNK1 phosphorylation, a corresponding reduction of PKCδ phosphorylation at the tyrosine 155 site was detectable in cells overexpressing FGFR2b Y769F (Fig. 4B). These findings suggest that in FGFR2b Y769F cells, the observed impairment of FGF7‐induced autophagy is due to the inhibition of PLCγ‐mediated signalling, which could involve the block of PKCδ activation and consequent JNK1 pathway shutdown.

**Figure 5:**
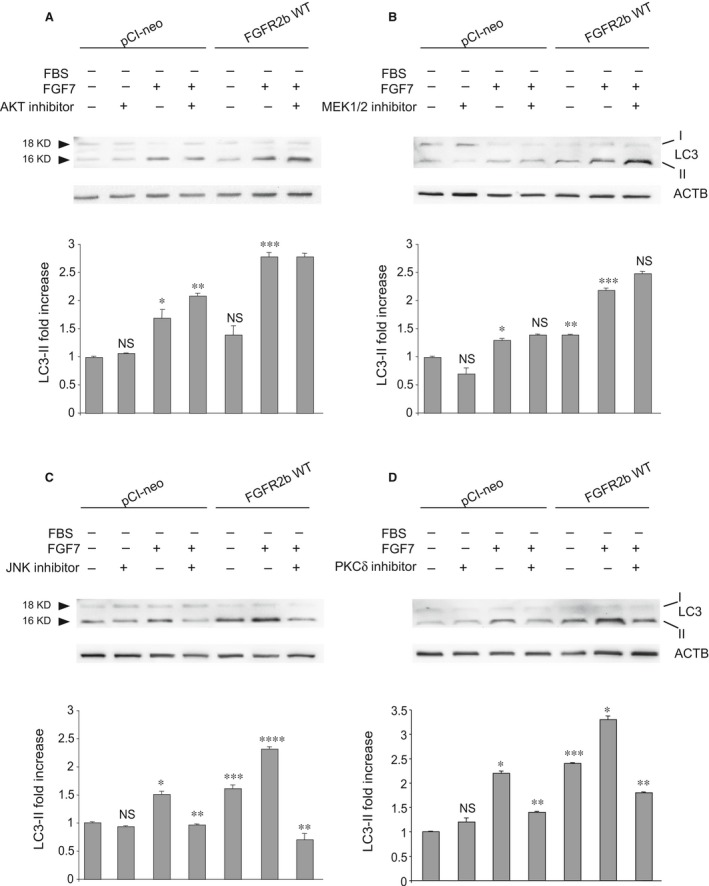
The inhibition of either JNK1 or PKCδ substrate blocks FGFR2b‐induced autophagy. (**A, B, C, D**) HaCaT pCI‐neo and HaCaT FGFR2b WT were serum‐starved and stimulated with FGF7 in the presence or absence of the indicated substrate inhibitors as reported in Materials and Methods. Western blot analysis shows that either AKT inhibitor (**A**) or MEK1/2 inhibitor (**B**) does not affect the increase in LC3‐II levels induced by FGF7, while JNK inhibitor (**C**) and PKCδ inhibitor (**D**) attenuate it. The equal loading was assessed with anti‐ACTB antibody. The densitometric analysis and Student's *t*‐test were performed as reported above: (**A**) *P <* *0.05 *versus* the corresponding FGF7‐unstimulated cells; **P <* *0.05 *versus* the corresponding FGF7‐stimulated cells; ***P <* *0.01 *versus* the corresponding FGF7‐unstimulated cells; NS *versus* HaCaT pCI‐neo cells. (**B**) *P <* *0.05 *versus* the corresponding FGF7‐unstimulated cells; **P <* *0.01 *versus* HaCaT pCI‐neo cells; ***P <* *0.01 *versus* the corresponding FGF7‐unstimulated cells; NS *versus* the corresponding MEK1/2 inhibitor‐untreated cells. (**C**) *P <* *0.05 *versus* the corresponding FGF7‐unstimulated cells; **P <* *0.01 *versus* the corresponding FGF7‐stimulated cells; ***P <* *0.01 *versus* HaCaT pCI‐neo cells; ****P <* *0.01 *versus* the corresponding FGF7‐unstimulated cells; NS *versus* the corresponding JNK inhibitor‐untreated cells. (**D**) *P <* *0.05 *versus* the corresponding FGF7‐unstimulated cells; **P <* *0.05 *versus* the corresponding FGF7‐stimulated cells; ***P <* *0.01 *versus* HaCaT pCI‐neo cells; NS *versus* the corresponding PKCδ inhibitor‐untreated cells.

To ascertain the relevance of JNK1 repression for the block of FGF7‐induced autophagy observed in cells expressing FGFR2b PLCγ signalling mutant, we estimated the impact of specific kinase inhibitors on LC3‐II levels in either HaCaT pCI‐neo or HaCaT FGFR2b WT. For JNK1, we took advantage of the JNK inhibitor (SP600125) also used to interfere with FGF18/FGFR4‐triggered JNK‐mediated autophagy 42, while the impairment of ERK1/2 signalling was obtained using the inhibitor of the upstream substrates MEK1/2, which efficiently block this pathway during ligand‐mediated activation of FGFRs 46. The effect of the AKT inhibitor previously described by us 10, 47 was checked again to further confirm that FGF7‐mediated autophagy is independent from the canonical PI3K/AKT/MTOR pathway. Western blot analysis performed with antibodies directed against the phosphorylated forms of each substrate confirmed that all the inhibitors were highly specific. In fact, AKT inhibitor selectively reduced AKT phosphorylation (Fig. S3A), while MEK1/2 inhibitor blocked the phosphorylation of the MEK substrates ERK1/2 (Fig. S3B). In addition, JNK inhibitor strongly impaired the Thr183/Tyr185 phosphorylation/activation in both JNK1 and JNK2 (Fig. S3C), and this effect, already previously documented, has been explained by the inhibition of the JNK autophosphorylating activity 48, 49. In the second step, we analysed the effects of the different substrate inhibitors on the LC3‐II modulation induced by FGF7 in either HaCaT pCI‐neo or HaCaT FGFR2b WT cells. Results showed that while both AKT and MEK1/2 inhibitors were ineffective (Fig. 5A and B), the JNK inhibitor clearly interfered with the increase in LC3‐II induced by FGF7 (Fig. 5C). As we have postulated that PKCδ could be a possible downstream PLCγ substrate acting upstream JNK1, we also analysed the effect of the direct inhibition of PKCδ on FGF7‐mediated autophagy. For this aim, we took advantage from rottlerin inhibitor, previously used to demonstrate the involvement of PKCδ in FGF/FGFR signalling pathways 45. Western blot analysis clearly showed that rottlerin exerted a repressive effect on FGF7‐induced LC3‐II increase (Fig. 5D) which was comparable to that induced by JNK inhibitor (Fig. 5C) or by PLCγ signalling shutdown in FGFR2b Y769F‐expressing cells (Fig. 3A). The efficiency of rottlerin was confirmed by its ability to induce a strong decrease in phosphorylation of the autophosphorylation site tyrosine 645 (tyrosine 643 in rat) 50 in PKCδ (Fig. S4); in addition, the evident repression of JNK1 phosphorylation (Fig. S4) confirmed the hypothesis that JNK1 is a downstream PKCδ substrate. Overall, our results suggest that the PLCγ signalling pathway is the main pathway responsible for the triggering of FGFR2b‐induced autophagy. The use of specific inhibitors confirmed that the autophagic process controlled by FGFR2b is PI3K/AKT/MTOR‐independent and requires JNK1 phosphorylation/activation, which possibly occurs *via* PKCδ activation.

### FGFR2b enhances FGF7‐mediated autophagy, phagocytosis and their convergence in light skin primary HKs

**Figure 6:**
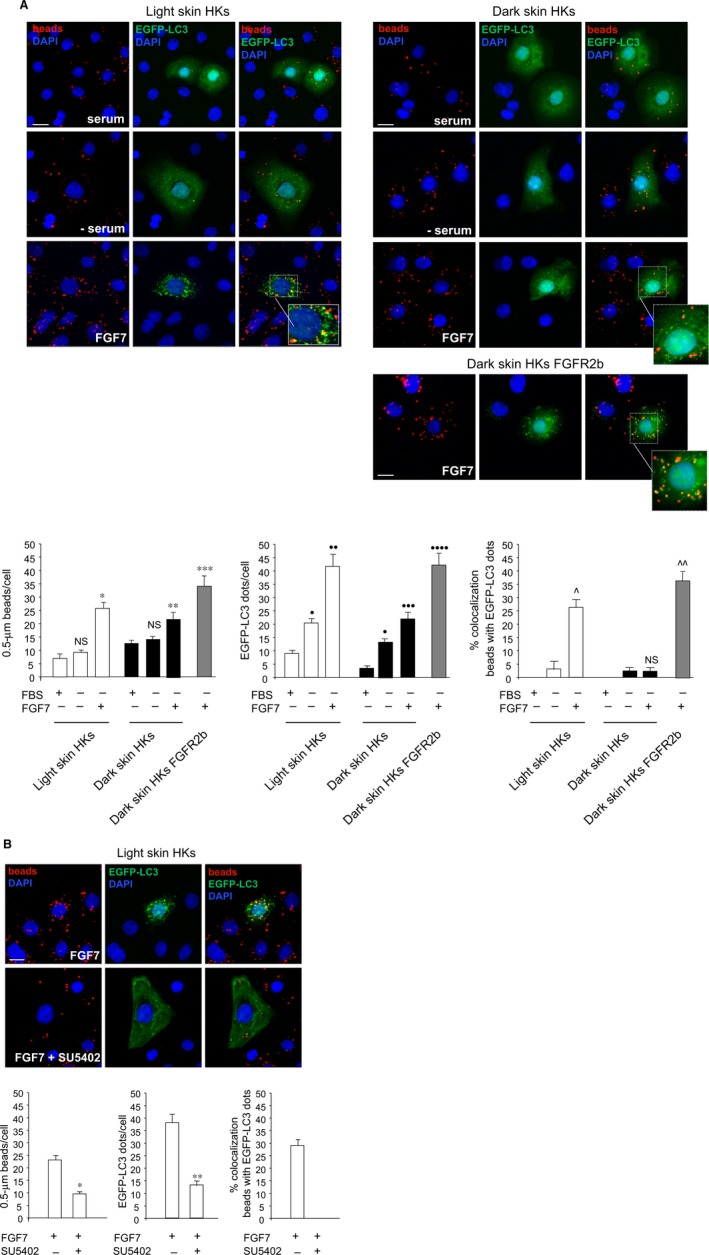
The efficiency of the convergence between FGF7‐induced phagocytosis and autophagy depends on FGFR2b expression. (**A**) HKs from light skin and from dark skin were transiently transfected with EGFP‐LC3 construct and serum‐starved and stimulated with FGF7 and incubated with fluorescent beads as reported in Figure 1. Alternatively, HKs from dark skin were transiently cotransfected with EGFP‐LC3 and FGFR2b (dark skin HKs FGFR2b). Quantitative fluorescence analysis shows that the increase in the bead uptake and the number of LC3‐positive dots per cell upon FGF7 stimulation is more evident in light skin HKs than in the dark ones. A partial colocalization between fluorescent beads and LC3 signal (26%) is detected only in light skin HKs. Dark skin HKs FGFR2b display a bead internalization, autophagosome formation and bead/LC3 colocalization comparable to those observed in light skin HKs. The quantitative analysis and Student's *t‐*test were performed as above: *P <* *0.01 *versus versus* the corresponding FGF7‐unstimulated cells; **P <* *0.05 *versus* the corresponding FGF7‐unstimulated cells; ***P <* *0.001 *versus* the corresponding untransfected dark skin HKs; NS *versus* the corresponding serum‐cultured cells; •*P *<* *0.01 *versus* the corresponding serum‐cultured cells; ••*P *<* *0.01 *versus* the corresponding FGF7‐unstimulated cells; •••*P *<* *0.05 *versus* the corresponding FGF7‐unstimulated cells; ••••*P *<* *0.01 *versus* the corresponding untransfected dark skin HKs; ^*P *<* *0.05 *versus* the corresponding FGF7‐unstimulated cells; ^^*P *<* *0.001 *versus* the corresponding untransfected dark skin HKs; NS *versus* the corresponding FGF7‐unstimulated cells. Bar: 10 μm. (**B**) HKs from light skin were treated with FGF7 and fluorescent beads as above in the presence of the FGFR2 inhibitor SU5402 as reported in Material and Methods. The bead uptake, the number of LC3‐positive dots per cell and the bead/LC3 colocalization are significantly reduced by SU5402. The quantitative analysis and Student's *t‐*test were performed as above: *P <* *0.05 *versus* the corresponding FGF7‐stimulated cells; **P <* *0.01 *versus* the corresponding FGF7‐stimulated cells. Bar: 10 μm

We have previously demonstrated that FGFR2b‐induced PLCγ signalling is required for FGF7‐stimulated phagocytosis and consequent melanosome uptake in keratinocytes 18; here, we demonstrated that the same signalling pathway is involved in FGFR2b‐mediated autophagy in these cells and that FGF7‐mediated phagocytosis and autophagy are converging pathways to possibly ensure melanosome degradation. In agreement with this possibility, in HKs from light skin, where FGFR2b‐mediated uptake of the melanosomes is higher than in HKs from dark skin as a consequence of an higher expression of FGFR2b 29, the reduced number of intracellular melanosomes was the consequence of an accelerated ability to degrade them by autophagy 51. In a recent work, we also speculated that in light skin HKs, FGFR2b, in virtue of its ability to accelerate the autophagosome turnover, could be responsible for melanosome removal *via* autophagy 10. On the basis of this hypothesis, we wondered whether FGFR2b could be involved not only in the regulation of both phagocytosis and autophagy, but also in the fine control of their balance. To ascertain it, we used again the *in vitro* model of bead uptake and we compared the ability of HKs from light and dark skin to either engulf or sort the engulfed beads to the degradation *via* autophagy in response to FGF7. To this aim, light and dark keratinocytes were transiently transfected with pEGFP‐C2‐LC3 construct, serum‐starved and treated with FGF7 and with inert latex red fluorescent beads as above. Quantitative fluorescence analysis performed as reported in Materials and Methods confirmed that as expected 29 and consistent with the increased expression of FGFR2b (Fig. S5), the treatment with FGF7 increased the bead uptake more in light skin keratinocytes than in the dark ones (Fig. 6A). In addition, in light skin HKs, FGF7 stimulation significantly increased the amount of EGFP‐LC3‐positive dots per cell more than in the dark ones and a part of beads (26%) colocalized with them (Fig. 6A). These results indicate that in light skin HKs, but not in the dark ones, the uptake of beads and their confluence in nascent autophagosomes for sorting to degradation are both enhanced by FGF7 stimulation. Interestingly, the transient transfection of dark skin HKs with FGFR2b (dark skin HKs FGFR2b) restored conditions comparable to those observed in light skin HKs in terms of bead internalization, autophagosome formation and bead/LC3 colocalization (Fig. 6A), confirming that the promotion of these events in response to FGF7 directly depends on FGFR2b expression. In addition, the effects observed in light skin keratinocytes in response to FGF7 appeared strongly dampened by the addition of the specific FGFR2 tyrosine kinase inhibitor SU5402 (Fig. 6B), indicating that FGFR2b activation and signalling are required.

## Discussion

The interplay between autophagy and phagocytosis is known to occur in the context of macrophages 12, 13, 14, 15, 16; however, a similar crosstalk has not been already described in other cell types. In this study, not only we confirmed our previous results showing that in human keratinocytes, FGF7 stimulation of its receptor FGFR2b is able to trigger both phagocytosis 18 and autophagy 10, but interestingly we found that the receptor activation is able to drive phagosomes and autophagosomes to converge and to reach the lysosomal compartment. This phenomenon might be explained as an additional strategy of this type of cells to more efficiently redirect the engulfed material towards a degradative fate.

It has been reported that in addition to the conventional autophagy, several non‐canonical autophagic mechanisms can be activated and, in particular, a direct recruitment of BECN1 and LC3 to the phagosomal membranes has been described in murine macrophages during phagocytosis and called LAP 16. However, our findings strongly indicate that in our cell model, the autophagic process mainly involves a canonical pathway to isolate the newly formed phagosomes. In fact, our fluorescence results, showing a not complete colocalization between the internalized beads and the LC3‐positive dots upon FGF7 stimulation, appear to exclude the involvement of a non‐canonical autophagy, such as LAP. This conclusion is further sustained by the results obtained through alternative depletion of components selectively required for autophagy or LAP pathways, such as ULK1 and Rubicon, respectively, and by the choice to use for our experiments inert latex beads, which do not *per se* induce LAP 30. In addition, our ultrastructural analysis strongly indicates that autophagosomal structures might close around phagosomes containing the beads and that this canonical intracellular membrane pathway may represent the main mechanism involved in such convergence of the two processes. However, several single‐membrane organelles containing beads were also visible; therefore, we may speculate that as previously described in macrophages during bacterial clearance 52, also in keratinocytes other mechanisms, including the fusion between the phagosomal membrane and the outer membrane of autophagosomes, cannot be excluded. Further work could be addressed to investigate whether FGF7 can interfere with LAP activated by specific stimuli, such as the treatment with zymosan or IgG beads 17, 30.

The ability of FGFR2b to regulate the phagocytic process appears to be functionally linked to the control of the melanosome uptake by recipient keratinocytes for skin pigmentation 18, 20. In fact, this ability in response to FGF7 is more efficient in HKs from light skin, which are known to express more FGFR2b compared to those from dark skin 29. In the other hands, a recent study highlighted the crucial role of the autophagic process in melanosome degradation in the context of human keratinocytes 51. Our present results, indicating a key role of FGFR2b not only in the induction of both autophagy and phagocytosis, but also in the regulation of their convergence, encouraged us to envisage a new scenario in which FGFR2b would differently control autophagy, and possibly melanosome turnover, in light and dark skin. This hypothesis appears to be strongly confirmed by our results obtained increasing the levels of FGFR2b expression in dark skin HKs through transfection: in fact, the forced expression of the receptor is not only able to enhance the bead uptake and the number of autophagosomes, but also promotes the autophagosomal/phagosomal convergence. This last evidence appears to unequivocally indicate that the divergent behaviour of HKs from differently pigmented skin in terms of melanosome uptake and clearance would be mainly ascribed to differences in FGFR2b expression. Taken together, our results suggest that through a fine modulation of the interplay between phagocytosis and autophagy, FGFR2b activation and signalling are crucial in determining melanosome intracellular amount and consequently skin pigmentation. Our speculations are consistent with those of Li and coworkers 53, which suggested that the UVB‐induced persistence of melanosomes, especially in HKs from light skin, can be due to the inhibition of autophagy consequent to UVB‐mediated internalization and degradation of FGFR2b 21, 54. These authors also proposed that the modulation of FGF7‐induced autophagy might be a useful strategy for treating skin pigmentation disorders 53.

**Figure 7:**
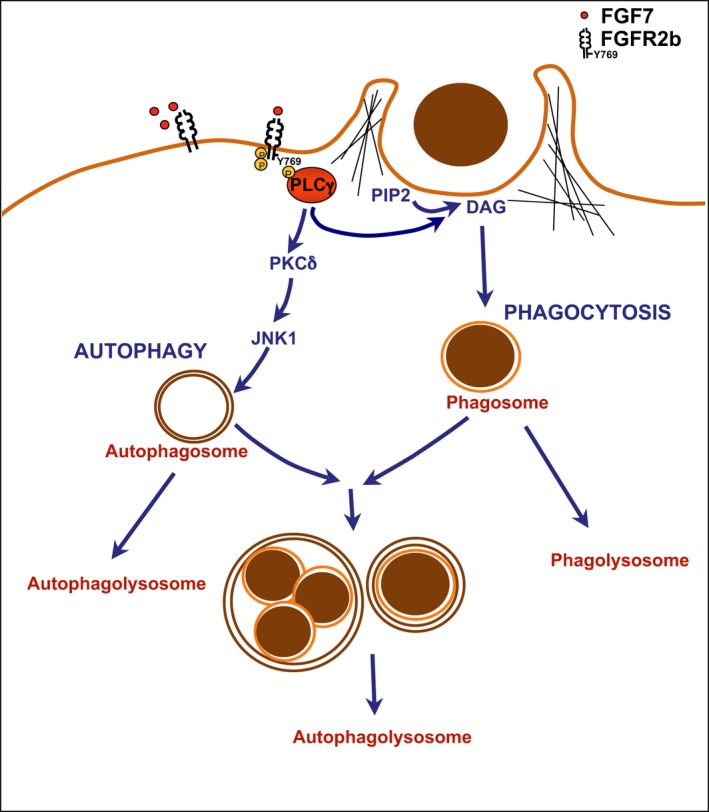
Schematic drawing of the proposed role of FGFR2b and its PLCγ signalling in the regulation of interplay between autophagy and phagocytosis. FGF7‐mediated FGFR2b activation induces phosphorylation of the Y769 residue, which is required for activation and recruitment of PLCγ to the receptor. PLCγ signalling in turn induces both phagocytosis through diacylglycerol (DAG) formation and autophagy through JNK1 activation *via* PKCδ. The two membrane pathways partially converge towards lysosomal degradation.

While in our previous work 18 we have demonstrated that in human keratinocytes, the FGFR2b‐induced phagocytosis occurs *via* PLCγ signalling, suggesting that diacylglycerol formation and consequent cortical actin reorganization could be required, the molecular pathways responsible for FGF7‐induced autophagy have not been clarified in our more recent studies 10, 11. It is well known that the autophagic process can be regulated by either MTOR‐dependent or MTOR‐independent molecular mechanisms 38, 39, and we have previously demonstrated that FGF7‐mediated autophagy is a PI3K/AKT/MTOR‐independent process 10. In this study, we further progress on the identification of the molecular mechanisms underlying FGF7‐mediated autophagy, identifying PLCγ as a crucial player acting downstream FGFR2b. Using biochemical and immunofluorescence approaches, we assessed the key role of the tyrosine 769 residue in FGFR2b, responsible for the activation/recruitment of PLCγ, not only in the receptor‐mediated phagocytosis as previously shown 18, but also in the induction of autophagy. In fact, the point mutation of this receptor site, which generates the PLCγ signalling dead mutant FGFR2b Y769F, resulted in the impairment of the autophagosome formation following FGF7 stimulation. Thus, PLCγ signalling appears to be the main pathway involved in the regulation of both FGFR2b‐mediated phagocytosis and autophagy (Fig. 7).

Then, to identify the molecular machinery acting downstream PLCγ, we focused our attention on JNK1, a RTK substrate able to activate an MTOR‐independent autophagic pathway 39 and recently identified as the main player involved in FGFR4‐mediated autophagy 42. We found that upon FGF7 stimulation, the phosphorylation/activation of JNK1 is strongly attenuated in cells overexpressing the FGFR2b Y769F mutant. Moreover, only the inhibition of JNK1, but not that of other FGFR2b substrates, such as MEK1/2 or AKT, was able to affect FGF7‐mediated autophagy. These findings demonstrated that consistent with what has been reported for FGFR4 in chondrocytes 42, FGFR2b‐mediated autophagy in keratinocytes is induced *via* the activation of JNK1 signalling. Taking advantage of the use of a specific inhibitor, we finally identified PKCδ as the molecular player downstream PLCγ 44 directly involved in JNK1 activation and consequently in FGF7‐induced autophagy. These results are consistent with previous findings indicating PKCδ as crucial activator of JNK1 pathway during autophagy induced by hypoxia 7, 43.

Overall, our data strongly suggest that PLCγ is the FGFR2b substrate, which acts as an upstream regulator of both phagocytosis and autophagy in HKs. While the PLCγ‐mediated formation of diacylglycerol and consequent cortical actin reorganization might be responsible for the triggering of phagocytosis, PKCδ/JNK1 signalling would be the main downstream PLCγ pathway required for the induction of FGF7‐mediated autophagy (Fig. 7).

## Conflict of interest

The authors declare no conflict of interests.

## Supporting information


**Figure S1** Uptake of 1 μm diameter beads in response to FGF7 stimulation.

**Figure S2** Tranfection with specific siRNAs induces efficient depletion of ULK1 and Rubicon proteins.

**Figure S3** Biochemical evaluation of the efficiency of different signaling pathway substrate inhibitors.

**Figure S4** Biochemical evaluation of the efficiency of PKCδ inhibitor.

**Figure S5** FGFR2b expression levels in HKs from differently pigmented skin.
