# Supplementary material for: Interplay between FGFR2b‐induced autophagy and phagocytosis: role of PLCγ‐mediated signalling
Source: J Cell Mol Med. 2017 Oct 10;22(1):668–83. doi: 10.1111/jcmm.13352 (PMC6193413; doi:10.1111/jcmm.13352)
Supplement: Supplementary file 1 — Figure S1 Uptake of 1 μm diameter beads in response to FGF7 stimulation. [file JCMM-22-668-s001.pdf]

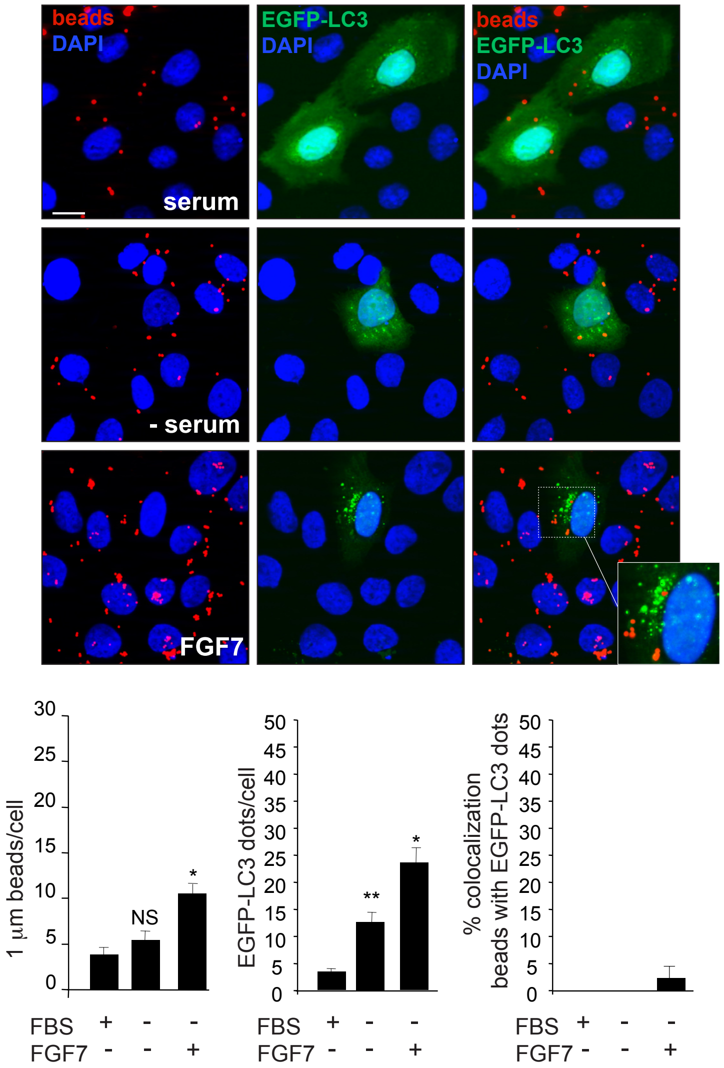

Uptake of 1µm diameter beads in response to FGF7 stimulation.

HaCaT cells were transiently transfected with pEGFP-C2-LC3 construct. Cells were then serum starved and stimulated with FGF7 for 24 h and with inert latex red fluorescent beads 1µm diameter for the last 4 h. Cell nuclei were stained with DAPI. Quantitative fluorescence analysis shows that bead uptake and the number of EGFP-LC3 positive dots are significantly increased by FGF7. Almost no colocalization between LC3 and fluorescent beads is detectable in response to FGF7. The quantitative analysis was performed as described in Materials and Methods and results are expressed as mean values ± standard errors (SE). Student t test was performed and significance levels have been defined as $p < 0.05$: *$p<0.01$ vs the corresponding FGF7-unstimulated cells; **$p<0.05$ vs the corresponding serum-cultured cells; NS vs the corresponding serum-cultured cells. Bar: 10 µm

Figure S1
