# Supplementary material for: Interplay between FGFR2b‐induced autophagy and phagocytosis: role of PLCγ‐mediated signalling
Source: J Cell Mol Med. 2017 Oct 10;22(1):668–83. doi: 10.1111/jcmm.13352 (PMC6193413; doi:10.1111/jcmm.13352)
Supplement: Supplementary file 2 — Figure S2 Tranfection with specific siRNAs induces efficient depletion of ULK1 and Rubicon proteins. [file JCMM-22-668-s002.pdf]

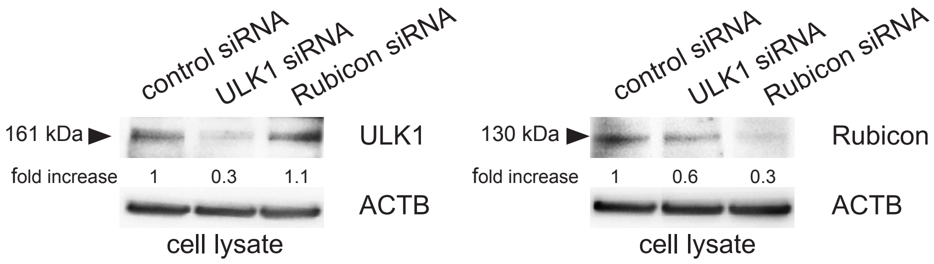

Tranfection with specific siRNAs induces efficient depletion of ULK1 and Rubicon proteins. HaCaT cells were transiently transfected with ULK1 siRNA, Rubicon siRNA or with an unrelated siRNA as control. Western blot analysis using anti-ULK1 polyclonal antibodies or anti-Rubicon monoclonal antibody shows that the band at the molecular weight corresponding to ULK1 protein is decreased in HaCaT ULK1 siRNA cells, while the band at the molecular weight corresponding to Rubicon is decreased in HaCaT Rubicon siRNA cells. The equal loading was assessed with anti-ACTB antibody. The densitometric analysis was performed as reported in Materials and Methods.

Figure S2
