# Supplementary material for: Interplay between FGFR2b‐induced autophagy and phagocytosis: role of PLCγ‐mediated signalling
Source: J Cell Mol Med. 2017 Oct 10;22(1):668–83. doi: 10.1111/jcmm.13352 (PMC6193413; doi:10.1111/jcmm.13352)
Supplement: Supplementary file 3 — Figure S3 Biochemical evaluation of the efficiency of different signaling pathway substrate inhibitors. [file JCMM-22-668-s003.pdf]

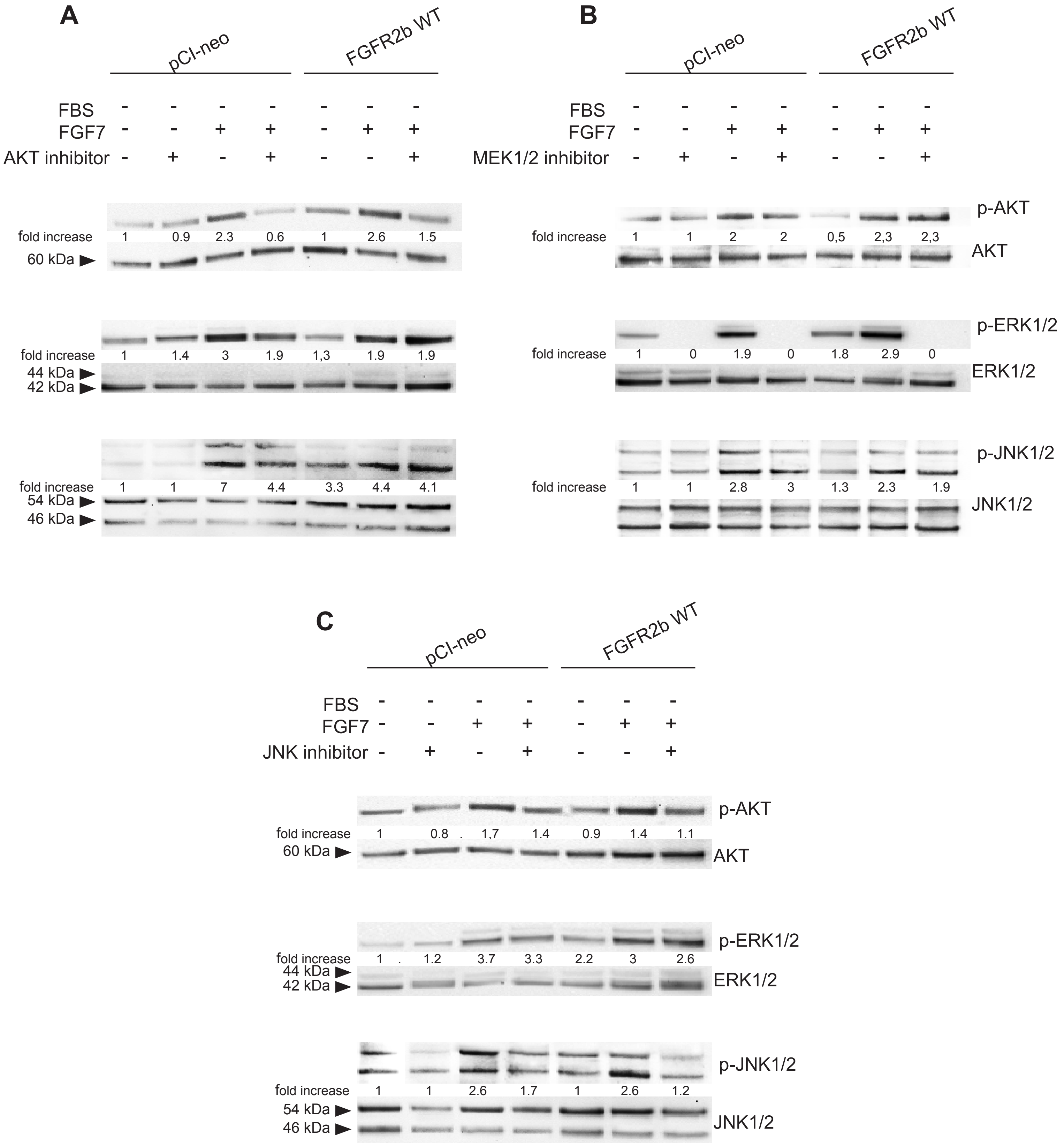

Biochemical evaluation of the efficiency of different signaling pathway substrate inhibitors. HaCaT pCI-neo and HaCaT FGFR2b WT cells were serum starved or stimulated with FGF7 in presence or not of the indicated substrate inhibitors as reported in Materials and Methods. Western blot analysis performed using antibodies directed against the phosphorylated forms of each substrate confirms that all the inhibitors were highly specific. The equal loading was assessed with anti-AKT, anti-ERK1/2 and anti-JNK1/2 antibodies. The densitometric analysis was performed as reported above.

Figure S3
