# Supplementary material for: Interplay between FGFR2b‐induced autophagy and phagocytosis: role of PLCγ‐mediated signalling
Source: J Cell Mol Med. 2017 Oct 10;22(1):668–83. doi: 10.1111/jcmm.13352 (PMC6193413; doi:10.1111/jcmm.13352)
Supplement: Supplementary file 4 — Figure S4 Biochemical evaluation of the efficiency of PKCδ inhibitor. [file JCMM-22-668-s004.pdf]

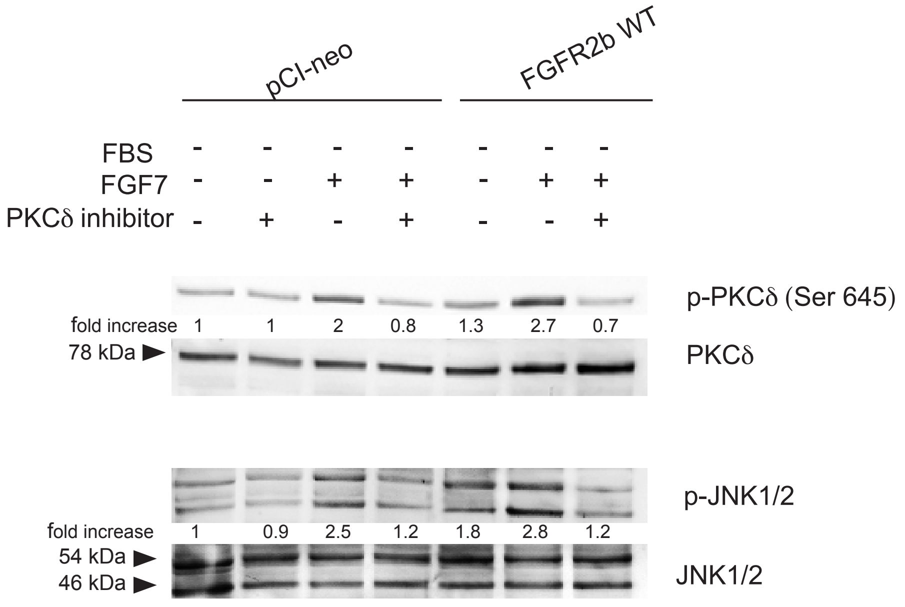


Biochemical evaluation of the efficiency of PKCδ inhibitor. HaCaT pCI-neo and HaCaT FGFR2b WT cells were serum starved or stimulated with FGF7 in presence or not of the PKCδ inhibitor as reported in Materials and Methods. Western blot analysis confirms that this inhibitor interferes either with the phosphorylation of the autophosphorylation site ser 645 in PKCδ and with the phosphorylation of JNK1 induced by FGF7 stimulation. The equal loading was assessed with anti-JNK1/2 and anti-PKCδ antibodies. The densitometric analysis was performed as reported above.

Figure S4
