# Supplementary material for: Interplay between FGFR2b‐induced autophagy and phagocytosis: role of PLCγ‐mediated signalling
Source: J Cell Mol Med. 2017 Oct 10;22(1):668–83. doi: 10.1111/jcmm.13352 (PMC6193413; doi:10.1111/jcmm.13352)
Supplement: Supplementary file 5 — Figure S5 FGFR2b expression levels in HKs from differently pigmented skin. [file JCMM-22-668-s005.pdf]

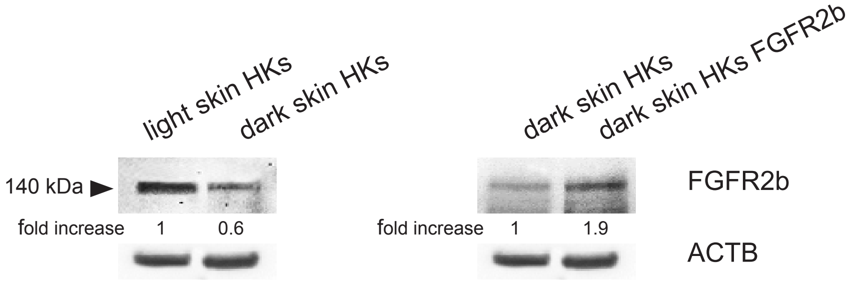

FGFR2b expression levels in HKs from differently pigmented skin. HKs from light skin and HKs from dark skin untransfected or transiently transfected with FGFR2b were grown in complete medium. Western blot analysis performed using anti FGFR2 polyclonal antibodies shows that a higher expression of FGFR2b is observed in light skin HKs and in dark skin HKs FGFR2b compared to untransfected dark skin HKs. The equal loading was assessed with anti-ACTB antibody. The densitometric analysis was performed as reported above.

Figure S5
